# Detecting Residual Awareness in Patients With Prolonged Disorders of Consciousness: An fNIRS Study

**DOI:** 10.3389/fneur.2021.618055

**Published:** 2021-07-28

**Authors:** Meng Li, Yi Yang, Yujin Zhang, Yuhang Gao, Rixing Jing, Yuanyuan Dang, Xueling Chen, Jianghong He, Juanning Si

**Affiliations:** ^1^School of Instrumentation Science and Opto-Electronics Engineering, Beijing Information Science and Technology University, Beijing, China; ^2^Department of Neurosurgery, Beijing Tiantan Hospital, Capital Medical University, Beijing, China; ^3^Brainnetome Center, Institute of Automation, Chinese Academy of Sciences, Beijing, China; ^4^National Laboratory of Pattern Recognition, Institute of Automation, Chinese Academy of Sciences, Beijing, China

**Keywords:** disorders of consciousness, functional near-infrared spectroscopy, motor imagery, support vector machine, minimally consciousness state

## Abstract

Recent advances in neuroimaging technologies have provided insights into detecting residual consciousness and assessing cognitive abilities in patients with disorders of consciousness (DOC). Functional near-infrared spectroscopy (fNIRS) is non-invasive and portable and can be used for longitudinal bedside monitoring, making it uniquely suited for evaluating brain function in patients with DOC at appropriate spatiotemporal resolutions. In this pilot study, an active command-driven motor imagery (MI) paradigm based on fNIRS was used to detect residual consciousness in patients with prolonged DOC. A support vector machine (SVM) classifier was used to classify yes-or-no responses. The results showed that relatively reliable responses were detected from three out of five patients in a minimally consciousness state (MCS). One of the patients answered all the questions accurately when assessed according to this method. This study confirmed the feasibility of using portable fNIRS technology to detect residual cognitive ability in patients with prolonged DOC by active command-driven motor imagery. We hope to detect the exact level of consciousness in DOC patients who may have a higher level of consciousness.

## Introduction

In recent years, considerable effort has been made to detect residual consciousness and to assess cognitive function in patients with absent or limited signs of consciousness. These patients are clinically diagnosed as having disorders of consciousness (DOC), including coma (unwakefulness, reflex behavior only), vegetative state/unresponsive wakefulness syndrome (VS/UWS), and minimally consciousness state (MCS) (1). Patients in VS/UWS are clinically awake but apparently unaware of themselves or their environment. In contrast, patients in MCS are partially aware of themselves and their environment and exhibit inconsistent but purposeful evidence of awareness, such as following verbal commands and visual pursuit (1, 2).

At present, behavioral assessments are the “gold standard” for detecting signs of awareness (2). However, because clinicians must depend on observable behaviors to determine the level of consciousness of patients with DOC (3), the rate of misdiagnosis was reported as ~40% (2). It is now well-accepted that a subset of residually aware patients will escape detection even after repeated and rigorous behavioral assessments by experienced teams (4). Because the sensitivity of standard behavioral testing is low, especially in patients with very limited movements, that is, those appearing to be in VS/UWS or low-level MCS (5), patients with a remarkable diversity of bedside examination and neuroimaging results are categorized as cognitive motor dissociation (CMD) patients (6). In other words, these patients are unable to show any behavioral signs of consciousness but may be able to respond mentally to active neuroimaging or electrophysiological paradigms (7). Therefore, in these patients, clear signs of awareness can be demonstrated using neuroimaging techniques that do not rely on an ability to produce an external response (8). A commonly used active paradigm is motor imagery (MI), which is the imagined movement of the body while keeping the muscles still (8, 9). The neural responses to a command can be a proxy for a motor action, so the responses can be interpreted as evidence of residual command following and, therefore, awareness. MI tasks include visual and kinesthetic tasks with kinesthetic MI having been reported to activate a greater proportion of the cortical motor system (2).

Advances in healthcare and neuroimaging technologies have provided insights into detecting residual consciousness and assessing cognitive abilities in patients with DOC (7). Several studies based on functional magnetic resonance imaging (fMRI) and electroencephalogram (EEG) have been conducted on the accurate detection of residual awareness and the diagnosis of patients with DOC to establish prognostic indicators and to explore the mechanism of consciousness (10). In 2006, Owen et al. asked a patient who had been clinically diagnosed as in a vegetative state to perform two MI tasks in a fMRI scanner; one was a tennis-playing imagery task and the other was a spatial navigation imagery task (11). The brain imaging evidence showed that the patient was actually conscious and had similar brain activities to healthy participants when completing the same tasks. Subsequently, several studies were conducted to assess the level of consciousness of patients with DOC (10). It has been reported that fMRI-based MI tasks could be used to obtain yes-or-no answers from some patients (12), implying the potential for brain–computer interfaces (BCIs) to establish basic communication with patients who appear to be unresponsive. Continuous EEG monitoring found that 15% of a group of patients with DOC had cognitive-motor separation (13). Recently, a new hierarchical auditory linguistic sequence paradigm including three processing levels was used to assess the depth of language processing in DOC patients and was able to distinguish between two different depths (14). This study found that speech-tracking neural responses and cortical dynamic patterns in DOC patients were directly associated with multiple levels of speech processing, providing the first EEG evidence that this active EEG paradigm can assist in the diagnosis and prognosis of DOCs. Despite the success of fMRI and EEG in investigating the brain activities in patients with DOCs, these techniques are somewhat limited. fMRI is cumbersome and expensive and cannot be used for longitudinal bedside monitoring. Moreover, fMRI scanners are sensitive to motion artifacts and unavailable for those with metallic implants. EEG technology has the advantages of high time resolution, safety, portability, and long-term continuous monitoring. However, the volume conductor effect greatly reduces its spatial resolution and limits its spatial positioning ability (15). Alternatively, functional near-infrared spectroscopy (fNIRS) (16–18) is an emerging non-invasive optical neuroimaging technology, which is portable, inexpensive, and wearable, has limited contraindications, and can be used for continuous repeatable monitoring in both natural and clinical environments. Moreover, fNIRS provides more information about the hemodynamic responses with improved ecological validity by measuring deoxygenated (HbR), oxygenated (HbO), and total (HbT) hemoglobin. However, studies assessing the cognitive ability detection of the residual consciousness of patients with DOC based on fNIRS are fragmented and limited so far. For example, Kempny et al. (19) demonstrated for the first time the feasibility of using fNIRS to evaluate the brain function of prolonged DOC (pDOC) patients using an MI paradigm. Abdalmalak et al. (20) verified the potential of an fNIRS-based MI task to communicate with a patient with locked-in syndrome, who, according to the fNIRS results, was able to provide correct answers to all questions. Therefore, in this pilot study, we used fNIRS to measure the real-time hemodynamic responses of patients with DOC using an active command-driven MI paradigm to study the brain functional activity of patients with DOC. The purpose of this study was to use an advanced active MI paradigm based on fNIRS to detect any credible higher level of consciousness that DOC patients may have.

## Materials and Methods

### Participants

The participants were recruited from the Department of Neurosurgery, 7th Medical Center of PLA General Hospital for this study. In this study, the inclusion criteria were (1) aged from 18 to 65; (2) etiology of traumatic brain injury, stroke, and anoxia, and duration of more than 28 days; (3) diagnosed as MCS according to the CRS-R scale; (4) intact auditory brainstem-evoked potentials that were confirmed by electrophysiological examination or behavioral inspection; and (5) able to obtain informed consent from the family members of the patients. The exclusion criteria were (1) diagnosed as VS according to the CRS-R scale; (2) other serious medical diseases or serious uncontrollable infections; (3) history of epilepsy, neurological, or psychiatric diseases; (4) severe aphasia or impaired cognition; and (5) inability to obtain informed consent.

**Figure 1:**
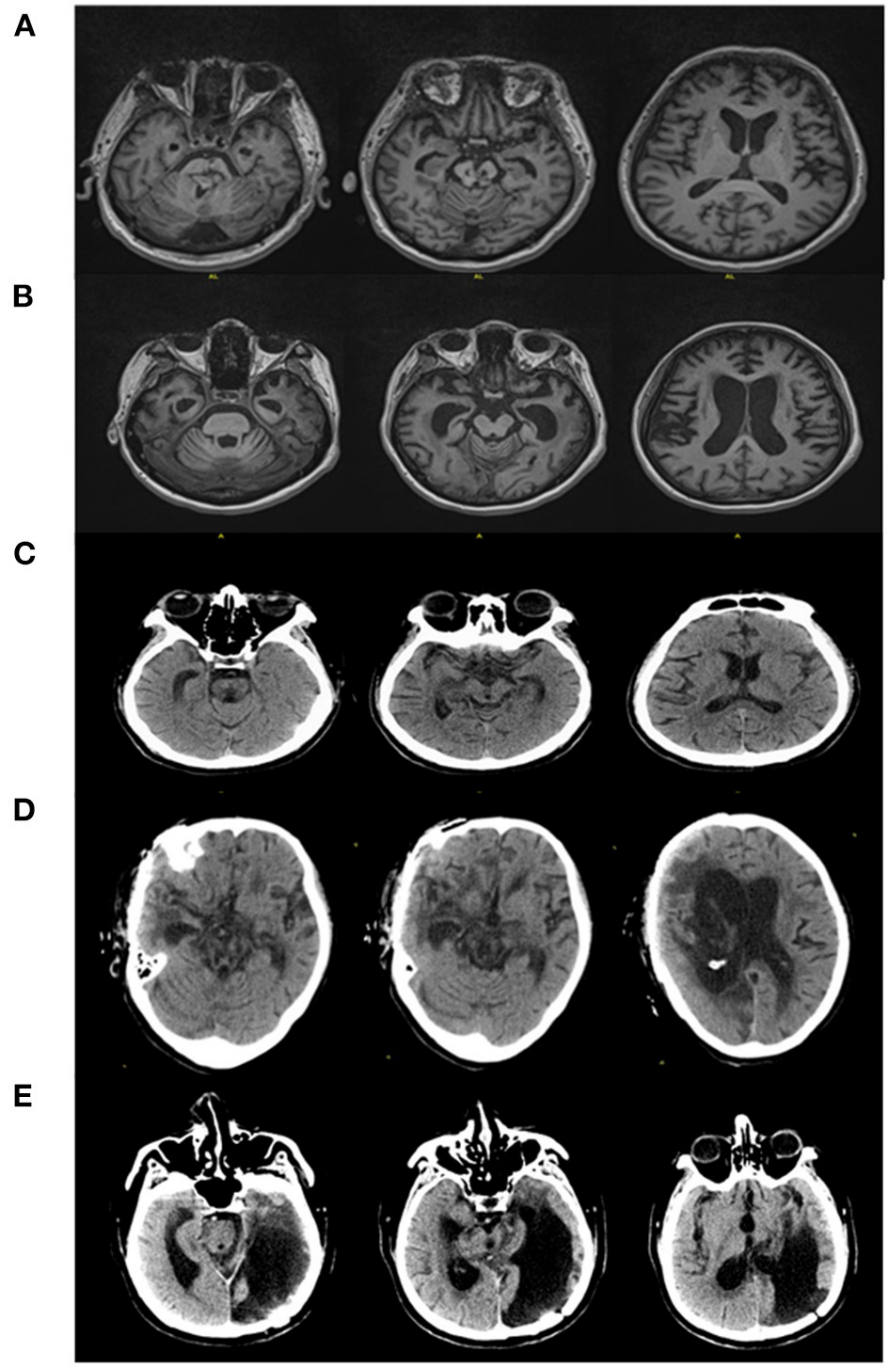
CT/MRI findings of patients. **(A)** MRI demonstrated brainstem malacia, brain atrophy (Patient 1). **(B)** MRI demonstrated whole-brain atrophy (Patient 2). **(C)** CT showed diffuse axonal injury, brainstem malacia (Patient 3). **(D)** CT showed postoperative changes of severe TBI, diffuse axonal injury, and brain stem malacia (Patient 4). **(E)** CT showed postoperative changes of severe TBI, brain stem malacia, and malacia of left occipital lobe and temporal lobe (Patient 5).

A total of 42 patients with DOC were recruited from June 1 to December 31, 2019, for this study. Since the active command-driven MI tasks required a higher level of consciousness to complete the tasks and due to the strict inclusion and exclusion criteria in the present study, only five MCS patients (four males and one female, ages 30–49 years) were finally selected for the MI tasks. The clinical characteristics of the patients are illustrated in Table 1. The consciousness level of these five MCS patients was evaluated before the experiment using the Coma Recovery Scale-Revised (CRS-R). The CT/MRI findings are shown in Figure 1. The control group consisted of six healthy subjects (four males and two females, ages 22–33 years). They were in good health and right-handed and had no history of any neurological or psychiatric diseases. Written informed consent for each subject in this study was obtained from the healthy subject or from the patients' legal guardians. The current study was approved by the ethics committee of the 7th Medical Center of PLA General Hospital.

**Table 1:** Clinical characteristics of patients with disorders of consciousness.

**No**.	**Diagnosis**	**Sex**	**Age (years)**	**Duration of DOC (months)**	**Etiology**	**CRS-R score**
1	MCS+	M	45	2	Stroke	17 (4–4–4–1–1–3)
2	MCS+	M	42	34	Anoxia	16 (3–4–5–1–0–3)
3	MCS-	M	41	19	TBI	10 (2–2–3–1–0–2)
4	MCS-	F	49	48	TBI	9 (2–1–3–1–0–2)
5	MCS-	M	30	2.5	TBI	8 (1–3–2–1–0–1)

*VS, vegetative state; MCS, minimally conscious state; CRS-R, Coma Recovery Scale-Revised, the CRS-R includes six subscales addressing auditory, visual, motor, oromotor, communication, and arousal functions, which are summed to yield a total score ranging from 0 to 23; TBI, traumatic brain injury*.

### Study Design

In the current study, the healthy control subjects and the patients with DOC were both required to perform kinesthetic MI tasks. The hemodynamic responses were detected by fNIRS from all the participants (six healthy control subjects and five patients) throughout the experiment. Because an experimental paradigm such as imagining hand squeezing or hand grasping may not be simple for DOC patients and the predictability of the patient's response is not necessarily high, the fNIRS-based active command-driven MI task was conducted according to the well-established “tennis imagery” (11) task, which requires subjects to imagine themselves playing a vigorous game of tennis.

The experiment consisted of two parts: first, the participant was required to perform the tennis imagery task to test his/her ability to successfully perform MI. This was confirmed by asking whether he could understand the experiment (Q1). Second, each subject was instructed to perform MI after responding to four questions: confirm his surname (correct) (Q2), if he has children for patients/confirm his surname (incorrect) for healthy subjects (Q3), confirm his father's name (Q4), and if there are four seasons in a year (Q5). The questions used in this study were selected based on the experience of the clinicians and questions utilized in previous studies (12, 20, 21). If the answer was “yes,” the subject was instructed to imagine himself playing a vigorous tennis game, swinging his arm to hit a tennis ball over and over again for 30 s; if the answer was “no,” the subject was asked to remain relaxed. The experimental paradigm was block-designed. Specifically, the entire experimental paradigm consisted of an initial baseline period (5 min) followed by five-question sessions.

**Figure 2:**
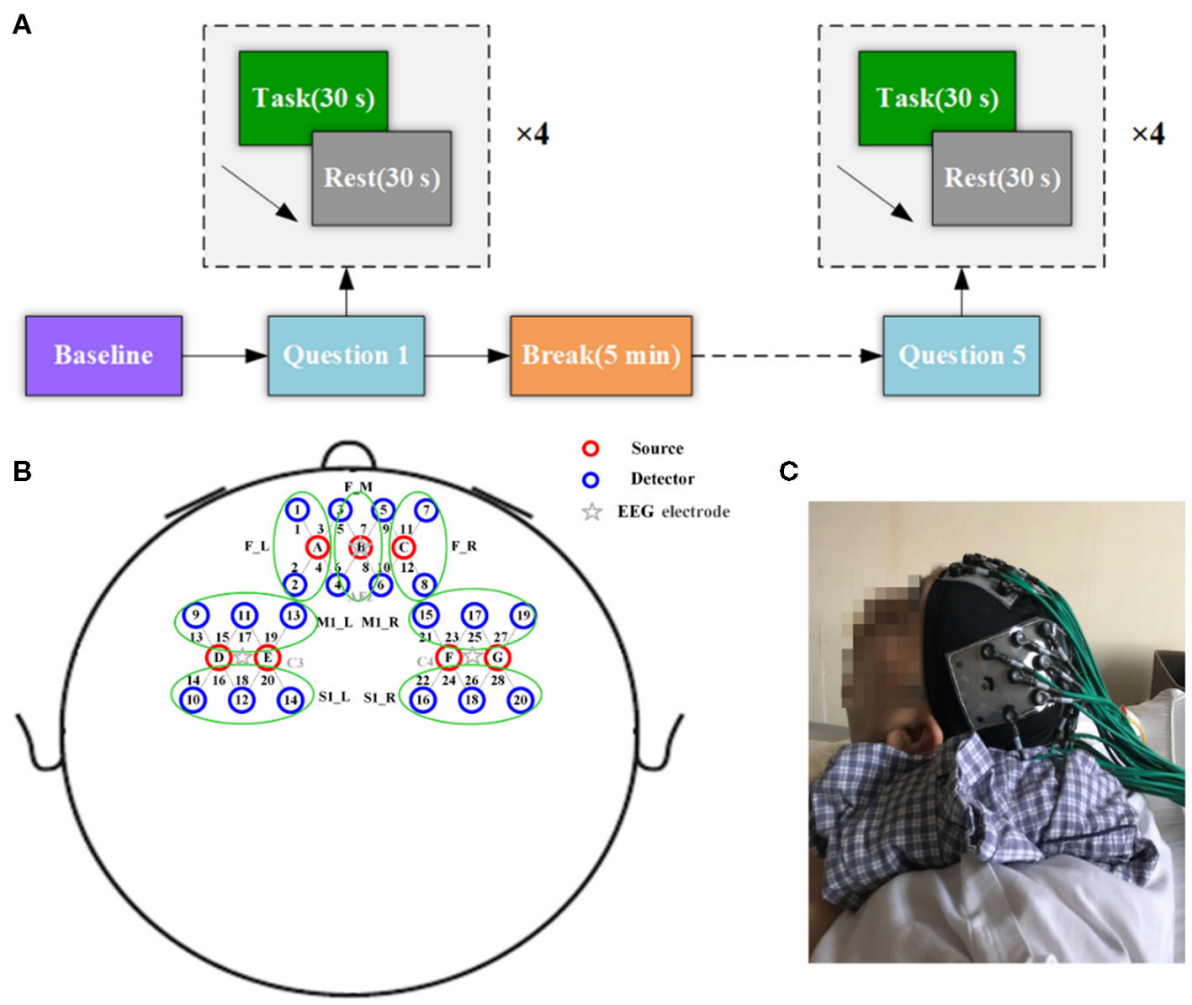
Experimental configuration. **(A)** Experimental paradigm of motor imagery tasks. **(B)** Arrangement of probes on the head. Specifically, seven sources (red circles) and 20 detectors (blue circles), yielding 28 optical channels (gray lines marked with channel numbers). **(C)** Photograph of the experimental setup.

Each question was repeated four times in a block design consisting of four cycles of alternating “task” (30 s) periods and “resting” (30 s) periods for a total duration of 4.5 min. To control the overall duration of the experiment and to minimize fatigue, each participant was given a 5-min break after each session. Before the formal experiment measurement, the experimental protocol was explained in detail to the participants so that they could become familiar with the experiment design. Auditory commands (“start answering” and “stay relaxed”) were used to indicate the onset of different actions. A schematic of the paradigm is presented in Figure 2A. For the patients with disorders of consciousness, the entire MI experiment was conducted with the assistance of a dedicated clinician. A photograph of the experimental setup is shown in Figure 2C.

### fNIRS Recording

The fNIRS data were acquired using the CW6 system (TechEn, Inc., Milford, MA, USA). The device emits light at two distinct wavelengths, 690 and 830 nm, for discrimination of two oxygenation states of tissue. In this study, the fNIRS optode pads were arranged above the prefrontal and motor cortex guided by the international 10–20 EEG electrode positions. Specifically, seven sources and 20 detectors were arranged geometrically over the prefrontal cortex (PFC), primary motor cortex (M1), and the primary somatosensory cortex (S1) to obtain 28 optical channels, as shown in Figure 2B. Specifically, the left and right optode pads were placed over the left and right motor-related areas, which centered on the C3 and C4 electrode positions, respectively. The frontal optode pad was placed over the PFC along the FP1–FP2 line, with the light source B located precisely at the AFz position. The distance between the source and detector pairs was 3 cm and covered an area of approximately 6 × 6 cm^2^ for each motor-related region and about 6 × 9 cm^2^ for the prefrontal region. The sampling rate for the fNIRS system was 50 Hz.

### Data Processing and Analysis

Data processing was conducted using Homer 2 software and the MATLAB 2013a platform (The MathWorks Inc., Natick, MA, USA). First, the original raw optical data were converted to the relative concentration changes of HbO, HbR, and HbT hemoglobin based on the modified Beer–Lambert law (MBLL) (22, 23). The differential pathlength factors (DPF) were 6.51 and 5.86 for 690 and 830 nm, respectively (24). Then, the data were bandpass filtered between 0.01 and 0.1 Hz to remove task-unrelated noise. Next, the data were segmented into epochs, starting 10 s before the activation onset and ending 20 s after the activation, and epochs with apparent artifacts (such as noise resulting from head motion) were rejected. After removing the noise, the block-averaged hemodynamic responses were calculated.

### Feature Selection and Classification

In this study, seven brain regions were measured based on the corresponding spatial positions, specifically F_L (channels 1–4), F_M (channels 5–8), F_R (channels 9–12), M1_L (channels 13, 15, 17, 19), M1_R (channels 21, 23, 25, 27), S1_L (channels 14, 16, 18, 20), and S1_R (channels 22, 24, 26, 28). During the data preprocessing, one healthy subject was excluded from further analysis because of poor data quality due to excessive motion artifacts. For the remaining 10 subjects, the average trial rejection rate was 13.8%.

For responses to the ground-truth “yes” questions from the normal subjects and the DOC patients, the channel number with the largest *t* value corresponding to each brain area was extracted, and the *t* value was defined as the mean value of HbO divided by the standard deviation of HbO within 5–35 s after the activation onset. The response to ground-truth “yes” questions was more obvious than that to ground-truth “no” questions and the signal-to-noise ratio was higher for responses to ground-truth “yes” questions. Therefore, for the ground-truth “no” questions, the hemoglobin features of the hemodynamic responses were obtained from the channel numbers that were used for the corresponding ground-truth “yes” questions. Once the channel number corresponding to each brain area was selected, the peak and mean values of HbO and HbR (Peak_HbO, Peak_HbR, Mean_HbO, Mean_HbR) over the range of 5–35 s after the activation were extracted as features for further analysis.

The responses to the ground-truth “yes” and “no” questions of the healthy subjects and the patients with impaired consciousness were classified using the LibSVM software package. The default RBF kernel function was used, and *x* represented the peak and average values for HbO and HbR obtained during the experiment for each brain area, *y*_*i*_represented the response type of the subject, and the label value +1 or −1 were respectively assigned to the positive and negative reactions. Subsequently, by optimizing the best parameters and using the model to predict a test set, the accuracies were calculated under two conditions: (1) the classification features used for the SVM classifier were extracted only from the motor area; (2) the classification features used for the SVM classifier were extracted from both the motor and prefrontal areas.

## Results

### Hemodynamic Results

**Figure 3:**
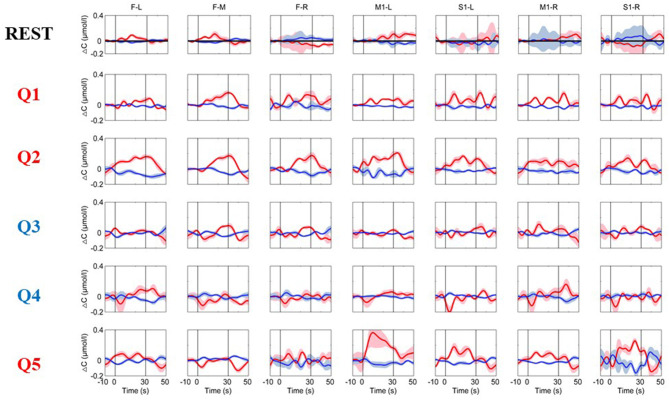
The time courses of the changes in HbO and HbR concentrations were obtained from a healthy subject (Subject 2). The resting-state time course labeled “REST” refers to data acquired without MI activation and is illustrated as a reference for comparison with the question periods. Q1–Q5 represents the hemodynamic responses for the different questions Q1–Q5. The red numbers represent the ground-truth “yes” questions, while the blue numbers represent the ground-truth “no” questions. The red and blue lines represent HbO and HbR, respectively. The stimulus duration is indicated by the space between the two gray lines.

**Figure 4:**
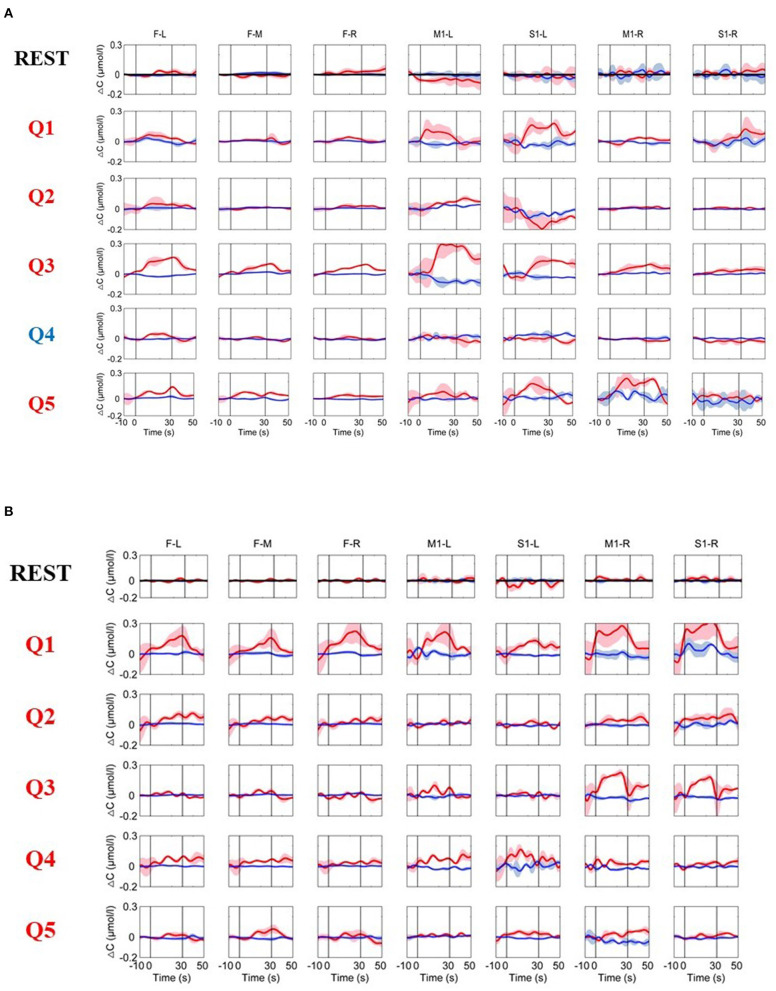
**(A)** The time courses of changes in HbO and HbR concentrations averaged across all three trials from a typical MCS patient (Patient 1). **(B)** The time courses of the changes in HbO and HbR concentrations from Patient 5. The resting-state time course labeled “REST” refers to data acquired without MI activation and is illustrated as a reference for comparison with the question periods. Q1–Q5 represent the hemodynamic responses for different questions Q1–Q5. The red numbers represent the ground-truth “yes” questions, while the blue numbers represent the ground-truth “no” questions. The red and blue lines represent HbO and HbR, respectively. The stimulus duration is indicated by the space between the two gray lines.

The time courses of the hemodynamic responses for a healthy subject are illustrated in Figure 3. To detect the functional activations for the MI tasks, the data during the resting state were used as the control. During the resting period, the hemoglobin concentrations over the prefrontal and motor areas were relatively stable. As shown in Figure 4, the distributions of the hemodynamic responses for the ground-truth “yes” questions (Q1, Q2, and Q5) and the ground-truth “no” questions (Q3 and Q4) were different. For example, the HbO concentration following the Q2 (“Confirming your surname”) question increased significantly, and the HbR concentration decreased correspondingly with smaller magnitudes during the task period over the prefrontal and motor areas, and their concentration gradually returned to the baseline after the task was over. Conversely, for the ground-truth “no” questions (Q3 and Q4), there were no noticeable changes in either the HbO or HbR concentrations. Similar patterns were observed in all five healthy subjects.

This method was subsequently used in patients with DOC, and reliable hemodynamic responses were detected from three out of five (Patient 1, Patient 2, and Patient 5) patients with DOC. The time courses of the hemodynamic response for a typical MCS patient (Patient 1) are shown in Figure 4A. The time courses of the hemodynamic response for an MCS patient (Patient 5) with a low CRS-R score but who showed significantly activated hemodynamic responses to the questions (note that the patient's five questions were all ground-truth “yes” questions) are shown in Figure 4B. For the resting-state data, the changes in the HbO and HbR concentrations were relatively stable and showed no significant changes in any of the brain areas. In comparison, the changes in the hemodynamic responses during the MI tasks were obviously different from the resting state. Specifically, for the ground-truth “yes” questions (Q1, Q2, Q3, and Q5 for Patient 1; Q1–Q5 for Patient 5), the HbO concentrations over the prefrontal and motor areas were relatively stable during the baseline period. After the task onset, the changes in HbO concentrations significantly increased during the task period for both the prefrontal and motor areas. Additionally, for the ground-truth “no” question (Q4), the changes in HbO concentrations were similar to the resting state. These dynamic patterns were highly consistent with the hemodynamic responses of the prefrontal and motor areas to an MI task stimulus in the normal subjects. In addition, for all five questions, the pattern produced always matched the factually correct answer.

**Figure 5:**
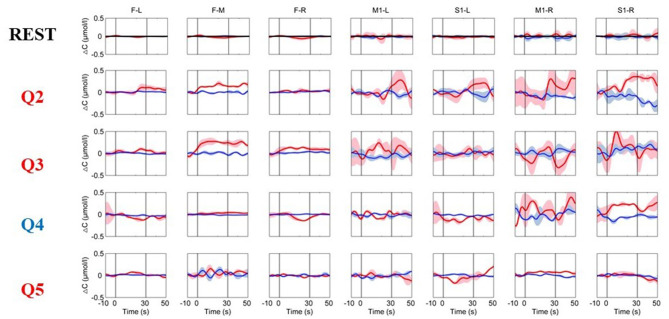
The time courses of changes in HbO and HbR concentrations were obtained from an MCS patient (Patient 3). The resting-state time course labeled “REST” refers to data acquired without MI activation and is illustrated as a reference for comparison with the question periods. Q2–Q5 represent the hemodynamic responses for different questions Q2–Q5. The red numbers represent the ground-truth “yes” questions, whereas the blue numbers represent the ground-truth “no” questions. The red and blue lines represent HbO and HbR, respectively. The stimulus duration is indicated by the space between the two gray lines.

The time course of the hemodynamic responses for an MCS patient (Patient 3) is illustrated in Figure 5. Question 1 had to be excluded due to significant motion artifacts. During the resting period, the hemoglobin concentrations over the prefrontal and motor areas were relatively stable. During the factual questions (Q2 and Q3), the HbO concentration in the prefrontal lobe and the motor area during the MI task sometimes increased or sometimes changed little or even decreased, and the responses were inconsistent. The fifth question showed a pattern that was very similar to the REST period; that is, there was no apparent response. For the question that was inconsistent with the facts (Q4), the PFC did not respond, but the HbO concentration in the channel located in the S1-R region unexpectedly increased. Therefore, no reliable and stable response was detected in this patient.

Most importantly, the distribution of hemodynamic responses during the MI period for three out of five patients with DOC was similar to those of the healthy control subjects, indicating that those patients could willfully modulate their brain activities to answer questions. No significant hemodynamic responses were measured for the other two patients with DOC.

### SVM Classification Results

To test the feasibility and reliability of fNIRS-based residual consciousness detection for patients with DOC using active command-driven motion imagery tasks, the classification was first conducted on the healthy control subjects. The SVM classifier was designed to classify the hemodynamic results under different conditions with different features. The classification results of the MI tasks for all the healthy control subjects are illustrated in Table 2. Specifically, as shown in Table 2, the classification accuracies differed for different conditions for the healthy control subjects. The fNIRS data over the PFC were also used as features for the SVM classifier. Specifically, when the peak HbO and mean HbO values over the motor (M1 + S1) area were used as features, the accuracy of SVM classification was 80%, whereas when both peak and mean values of HbO and HbR over the motor (M1 + S1) areas were used as features, the accuracy increased to 90%. Note that if the features over the prefrontal were further used for SVM classification, the accuracy was consistently 90%.

**Table 2:** Classification accuracies of the fNIRS-based motor imagery task for the healthy control subjects.

**Features**	**M1 + S1 accuracy (%)**	**PFC + M1 + S1 accuracy (%)**
Peak_HbO, Mean_HbO Peak_HbR, Mean_HbR	90	90
Peak_HbO, Mean_HbO	80	90

After establishing that this method yielded reliable and feasible results, functional communication with a patient with MCS (Patient 1) was tested using active command-driven MI tasks with the response assessed by fNIRS. As shown in Table 3, the classification results of the MI tasks for the patient with MCS was consistently 100%.

**Table 3:** Classification accuracies of the motor imagery task for a typical patient with MCS (Patient 1).

**Features**	**M1 + S1 accuracy (%)**	**PFC + M1 + S1 accuracy (%)**
Peak_HbO, Mean_HbO Peak_HbR, Mean_HbR	100	100
Peak_HbO, Mean_HbO	100	100

## Discussion

The clinical detection of residual consciousness and assessment of cognitive ability is challenging for clinicians evaluating patients with DOC. At present, behavioral assessments are the “gold standard” for evaluating the signs of awareness. Because such behaviors are often subtle, inconsistent, and fluctuating, behavioral assessments are susceptible to misdiagnosis (25). In recent years, advances in neuroimaging techniques have significantly promoted the assessment of consciousness in patients with DOC.

In this study, an fNIRS-based active MI paradigm was used to identify residual brain activation in patients with DOC. To test the feasibility and reliability of this method, healthy control participants were asked to kinesthetically imagine the same task. For the healthy control subjects, the distributions of hemodynamic responses evoked by the MI tasks for the ground-truth “yes” questions were different from those of the ground-truth “no” questions. Specifically, for the ground-truth “yes” questions, the HbO concentrations significantly increased after the activation onset compared to the baseline period, whereas for the ground-truth “no” questions, no significant hemodynamic responses were found. After establishing this response pattern, the MI tasks were further conducted with the patients with MCS. The distribution patterns of the hemodynamic responses of the patients with MCS were further compared with those of the healthy control subjects. The result was that the distribution patterns of the hemodynamic responses in three out of five patients with MCS were similar to those in the healthy control subjects, indicating that those three patients with MCS could willfully modulate their brain activation to try to answer the simple “yes-or-no” questions through the fNIRS-based MI tasks. Note that one MCS patient (Patient 1) was able to produce completely reliable neurological responses and could give the correct answers to all five questions. Interestingly, although another patient with MCS (Patient 5) had a relatively low CRS-R score, the fNIRS results showed similar distributions of the hemodynamic responses. This finding may indicate that this patient has a higher level of consciousness than the CRS-R score would indicate. Compared with the subjective behavioral assessment, evaluation by fNIRS detected the residual consciousness of patients with DOC objectively and may provide some insights for clinicians in the diagnosis, treatment, and prognosis of patients with DOC. Moreover, this study is a preliminary feasibility verification study; after verifying the feasibility and reliability of this method on the MCS patients, further studies with advanced technologies and experimental paradigms will be conducted on different kinds of DOC patients, such as UWS and MCS patients. We hope to figure out the differences between the MCS and VS patients and provide new insights into the diagnosis of DOC patients.

It has been reported that SVM can achieve a better balance between sensitivity and specificity than linear discriminant analysis (LDA) methods (21, 26). With the caveat that our research was a small-sample pilot study, the SVM classifier appears to be suitable for classifying hemodynamic responses under different conditions. In this study, the accuracy of the healthy people's M1 + S1 motor area was 90%, which is higher than a previous study using SVM as a classifier to classify healthy people's MI tasks (76%) using a four-channel TR-NIRS system to interrogate the SMA and PMC (21). The accuracy of one MCS patient (Patient 1) was also comparable to the result obtained for a DOC patient who did communication scanning in an fMRI study (12). Furthermore, our results showed that when the classification features increased, the classification accuracy of the normal participants showed a significant improvement (Table 2). When the peak_HbO and mean HbO values over the motor (M1 + S1) areas were used as features for the SVM classification, the accuracy was 80%, but when the peak HbR and mean HbR values over the motor (M1 + S1) areas were added as features, the accuracy increased from 80 to 90%. This phenomenon indicates that HbR data can provide additional information that is useful for classification. Previous studies have also shown that an improved performance could be achieved by utilizing more informative features or classifiers through a more detailed inspection of the activation patterns or a better selection of motor tasks (2). Therefore, increasing the amount of feature information may help improve classification accuracy.

As a pilot study, the current results showed that residual consciousness can be detected in patients with DOC using fNIRS-based active MI tasks. Our research also has some limitations. The small sample size is one limitation. Because this special group had so many requirements for participation and given that command-driven MI tasks require a high level of consciousness to complete the tasks, it was difficult to obtain a large number of samples in a short period. Although the sample size was relatively small, the main purpose of this pilot study was to preliminarily verify whether the emerging non-invasive, portable, optical imaging technology (fNIRS) could be used as a reliable tool for detecting residual and active consciousness in patients with disorders of consciousness (DOC). In further studies when we can obtain more patients and improve the fNIRS technique, the findings of this study should be tested to estimate its reliability and reproducibility so that practitioners can further expand its use in the clinical environment. A second limitation is that only the “tennis playing” MI strategy was used in this study. However, there is still a lack of a control condition for other MI strategies, including the “hand squeezing (27)” MI task, so different MI strategies may lead to different patterns of task-evoked hemodynamic responses. In further studies, different types of MI strategies such as “tennis playing” and “hand squeezing” will be used to quantitatively compare the different hemodynamic responses of the DOC patients during a variety of command-driven MI tasks. The third limitation is that five questions were used; this is a relatively small number to fully investigate the residual consciousness of patients with DOC. Currently, fMRI, EEG, and fNIRS technologies all have technical deficiencies in terms of specificity and sensitivity in the detection of brain consciousness. That is, the detection of command-following activities can basically confirm that there is residual consciousness, but the failure to detect that a patient is following the activities does not mean that there is no consciousness. In further studies, an advanced experiment paradigm with more questions should be conducted to provide more information about the brain function of patients with DOC.

Although there are certain limitations, this pilot study provides insights into evaluating the residual cognitive function of patients with DOC. We hope that this study can provide some useful information that could eventually be used to construct a BCI as a communication tool for patients with DOC. With the development of neuroimaging and BCI technologies, in the future the residual cognitive ability of patients with DOC can be better evaluated. It is also possible that BCI could eventually be used to grade the differences in cognitive abilities of these patients. However, before that can occur, the residual cognitive function of these patients must be better understood so that eventually these augmentative communication technologies can be successfully implemented. It is clear that the current findings are just the tip of the iceberg. There is still much to do to ensure that fNIRS data is sufficiently reliable for use in clinical communication with patients with DOC. As mentioned above, fNIRS has the potential to play a vital role in assessing the cognitive ability of patients with DOC.

## Data Availability Statement

The data analyzed in this study is subject to the following licenses/restrictions: The data involves the privacy of the patients, so it is not convenient to provide the original data. Requests to access these datasets should be directed to Juanning Si, sijuanning@bistu.edu.cn.

## Ethics Statement

The studies involving human participants were reviewed and approved by the 7th Medical Center of PLA General Hospital. The patients/participants provided their written informed consent to participate in this study. Written informed consent was obtained from the individual(s) for the publication of any potentially identifiable images or data included in this article.

## Author Contributions

ML was mainly responsible for collecting data, processing and analyzing data, and writing papers. YY and JH were responsible for clinical technical guidance. YG was responsible for providing relevant information and data analysis. XC and YD were responsible for assisting in data collection. JS and RJ were responsible for designing experiments, directing experiments, and revising papers. All authors contributed to the article and approved the submitted version.

## Conflict of Interest

The authors declare that the research was conducted in the absence of any commercial or financial relationships that could be construed as a potential conflict of interest.

## Publisher's Note

All claims expressed in this article are solely those of the authors and do not necessarily represent those of their affiliated organizations, or those of the publisher, the editors and the reviewers. Any product that may be evaluated in this article, or claim that may be made by its manufacturer, is not guaranteed or endorsed by the publisher.
